# Relationship between the actual fine dust concentration and media exposure that influenced the changes in outdoor activity behavior in South Korea

**DOI:** 10.1038/s41598-020-68580-4

**Published:** 2020-07-20

**Authors:** Myung-Gwan Kim, Su-Jin Lee, Donghwi Park, Chul-hyun Kim, Ki- hoon Lee, Jong-moon Hwang

**Affiliations:** 1 Graduate School of Public Health, Kyungpook National University, Daegu, South Korea; 2 Department of Physical Medicine and Rehabilitation, Ulsan University Hospital, Ulsan, South Korea; 3 University of Ulsan College of Medicine, Dong-gu, Ulsan, South Korea; 4 Department of Rehabilitation Medicine, Kyungpook National University Hospital, 200 Dongduk-ro Jung-gu, Daegu, 700-721 South Korea; 5 Department of Rehabilitation Medicine, School of Medicine, Kyungpook National University, 200 Dongduk-ro Jung-gu, Daegu, 700-721 South Korea; 6 Mompyeonhan Rehabilitation Clinic, Daegu, South Korea

**Keywords:** Public health, Climate-change impacts

## Abstract

The one reason of the decrease of walking time for adults in South Korea among various factors is the sense of fear about fine dust sparked by media reports, which has created a negative perception of fine dust. This study aimed to assess the change in concentration of fine dust, as well as individuals' walking time and health status, in South Korea, and to investigate the relationship between the media reports on fine dust. Using the national government statistics data, we analyzed the relationship between walking time, concentration of fine dust, and amount of media reports on fine dust. From 2008 to 2017, the average walking time and PM10 levels decreased from 76.17 to 49.47 min and 52 to 45 μg/m^3^; whereas PM10 media frequency increased from 349 to 9,234. No positive correlation existed between walking time in South Korea and exposure to fine dust. However, media reports on fine dust increased steadily from 2012 and peaked in 2015. The decrease in average walking time in South Korea was due to the negative perception created by the increase in media reports on fine dust, rather than the increase in the actual concentration of fine dust.

## Introduction

Interest in particulate matter (PM) among South Koreans is increasing daily. Concerned with the increase in concentration of fine dust and the resultant deterioration of the health of citizens, the Ministry of Environment of South Korea promulgated enforcement regulations “Special Act on Fine Dust Reduction and Management” in February 2019^1^. The purposes of the regulations are to reduce the emission of fine dust and the product of pollutants, manage the occurrence of such pollution to prevent the hazards associated with fine dust, preserve the atmosphere of the environment in an optimal state, and set up a comfortable living environment.

The argument has been amplified through newspapers, news, and various social media networks as a result of the ‘Fine Dust Emergency Mitigation Measure’, changes in the environment, and changes in the health status of individuals. Therefore, the negative perceptions and inconveniences of the people have increased considerably^2^. The public’s perception of fine dust is expressed in negative emotional terms, they are expressed together with terms with negative meaning, which are related to government activities^3^. These invisible risk issues cause citizens to have uncertainties and fears about fine dust itself, and they are as well dependent on the media for information on ways to protect themselves from these invisible risks^4^.

The National Institute of Environmental Research (NIER) of South Korea announced that fine dust occurs in boilers, automobiles, factor operation facilities, etc., and these sources account for a considerable amount of fine dust measured domestically. That is, a considerable part of fine dust generation can be attributed to domestic factors^5^. Analysis of the contributions of fine dust domestically and from foreign countries in 2018 by NIER revealed that the average foreign contribution constituted about 44–55%, which is different from the 60–80% of high concentration of fine dust known to be contributed by foreign influences^6^. It is supported by the facts that much of fine dust generation is due to domestic factors that are a continuation of domestic high pressure impact and stagnation of Barometer^7^, increased direct air pollutant emissions domestically^8,9^, and difference between measurement sites due to emitted air pollutants cannot move away^10^ even if high concentration fine dust in the windward area such as Chinese Bohai region formed the inflow route to the South Korea (domestic) by the northwest wind^11,12,13^.

In the analysis of fine dust-related keywords in “Big Kinds”, a comprehensive news database, provided by the South Korea Press Foundation-News Big Data & Analysis System, some articles with business purposes, such as specific services or household goods, were related with fine dust in spite of media coverage. These articles use short-term strategies from focused on simple interest that are not based on fundamental problem-solving methods, and aimed at increasing the public’s response to fine dust. However, these short-term strategies used in media reports on fine dust, which actually requires a long-term strategy, appear to have negative influences on policy making and the utility^14^. In one study^15^ comparing the variation in fine dust concentration in South Korea by air quality evaluation reports^16^ and the frequency of media reports related to fine dust concurrently, an explosion of media coverage of PM, caused the implementation of the fine dust forecasting system, according to the Ministry of Environment’s revised < the Air Quality Preservation Act > in July 2013. However, no significant association existed between the actual increase in PM. In contemporary societies, the most frequently used model for modeling various social risk factors is the Social Amplification of Risk Framework^17^. In Kim’s study, the model revealed that public perception of risk phenomenon in modern societies depends on social and cultural characteristics, which can determine the public’s perception and behavior change according to the risk phenomena^18^. The risk that the public are aware of the fine dust shall be two kinds exist: “objective risk’ related to physical damage and ‘subjective risk’ related to the object’s perception, regardless of actual damage^14^. Especially, since knowledge of the danger of fine dust is transmitted to the public by the media and spread to the public, it is recognized as a ‘subjective risk’ to the public. So the importance of proper media coverage of fine dust is being emphasized.

The purpose of this study was to assess the changes in the actual concentration of fine dust over recent years, analyze the health status related to behavior index of the public, and identification of the risk factors. This research was conducted on the hypothesis that a positive correlation exists between the variation in actual concentration of fine dust during this period and the health status and health-related behavior index of the public. Using the Korea National Nutrition Examination Survey, if the health-related behavior index of the public decreases, it is due to an increase in excess media report on fine dust to understand the relationship between various external, domestic-foreign, urban-environmental, and socio-environmental factors. Based on this research, it is necessary to change the perception of factors that negatively affects public health behavior by creating a fear of fine dust among the public, which is as a result of indiscreet media coverage on fine dust. This study did not only seek to identify the sources of fine dust inflow from outside factors but also to reduce the sources of fine dust by suggesting various possible domestic and external factors. In addition, we hope that it can be used as basic data to emphasize the necessity of comprehensive health promotion and policy support.

## Method

### Research on composite data using national government statistics

**Figure 1:**
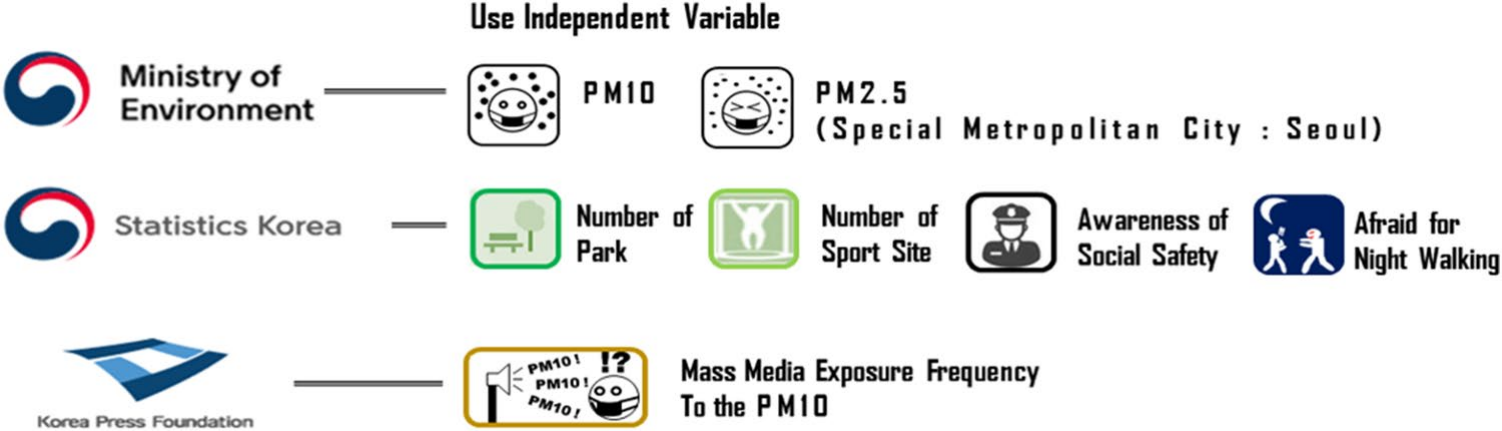
Research on composite data using national government statistics. We used a variable factor to analyze the changes in actual fine dust concentration, health behavior, index related health status and frequency of media reports related to fine dust from 2008 to 2017. Republic of Korea Environmental Statistics Yearbook Data for PM (particulate matter) 10 and PM2.5 in Seoul City. Republic of Korea Ministry of Statistics Social Survey Data for awareness of social safety, afraid for night walking. Republic of Korean Ministry of Statistics Korean City and Province Basic Statistics Data for number of park and number of sport site. Republic of Korea Press Foundation is open provide social mass media exposure data in news big data & analysis system for mass media exposure related particulate matter.

These research data were derived from the national government statistics, which is a merger of data from the National Health and Nutrition Examination Survey, Environmental Statistics Yearbook, Ministry of Statistics Social Survey, Ministry of Economy and Finance, and Press Foundation-News Big Data & Analysis System: The Big Kinds-Mass Media Exposure Data. These data are merged based on city codes of cities in South Korea. This was an ecological model and trends study in the decade from 2008 to 2017 (Fig. 1).

### National health and nutrition examination survey data

The National Health and Nutrition Examination Survey^19^ is a statutory survey on the health behaviors of the public, current status of chronic diseases, and actual condition of food and nutrition based on Article 16 of the National Health Promotion Act. The data of the National Health and Nutrition Survey of South Korea was collected through the circular sample survey design of the citizens whose subjects themselves could represent South Korea. In addition, when this data is provided to researchers, there is no problem of personal information leakage because the personal information is statistically secured. In other words, there was no problem of research ethics or sampling during analysis.

The National Health and Nutrition Examination Survey was conducted every 3 years from the first period (1998) to the third period (2005). Thereafter, it was reorganized as a yearly survey system and was carried out annually during the fourth period (2007–2009). The survey was conducted with the approval of the Research Ethics Review Committee of the Disease Control Division, and the results of the survey were published through the dissemination of press releases, statistical publications, and publication of the original data until December of the next year.

**Figure 2:**
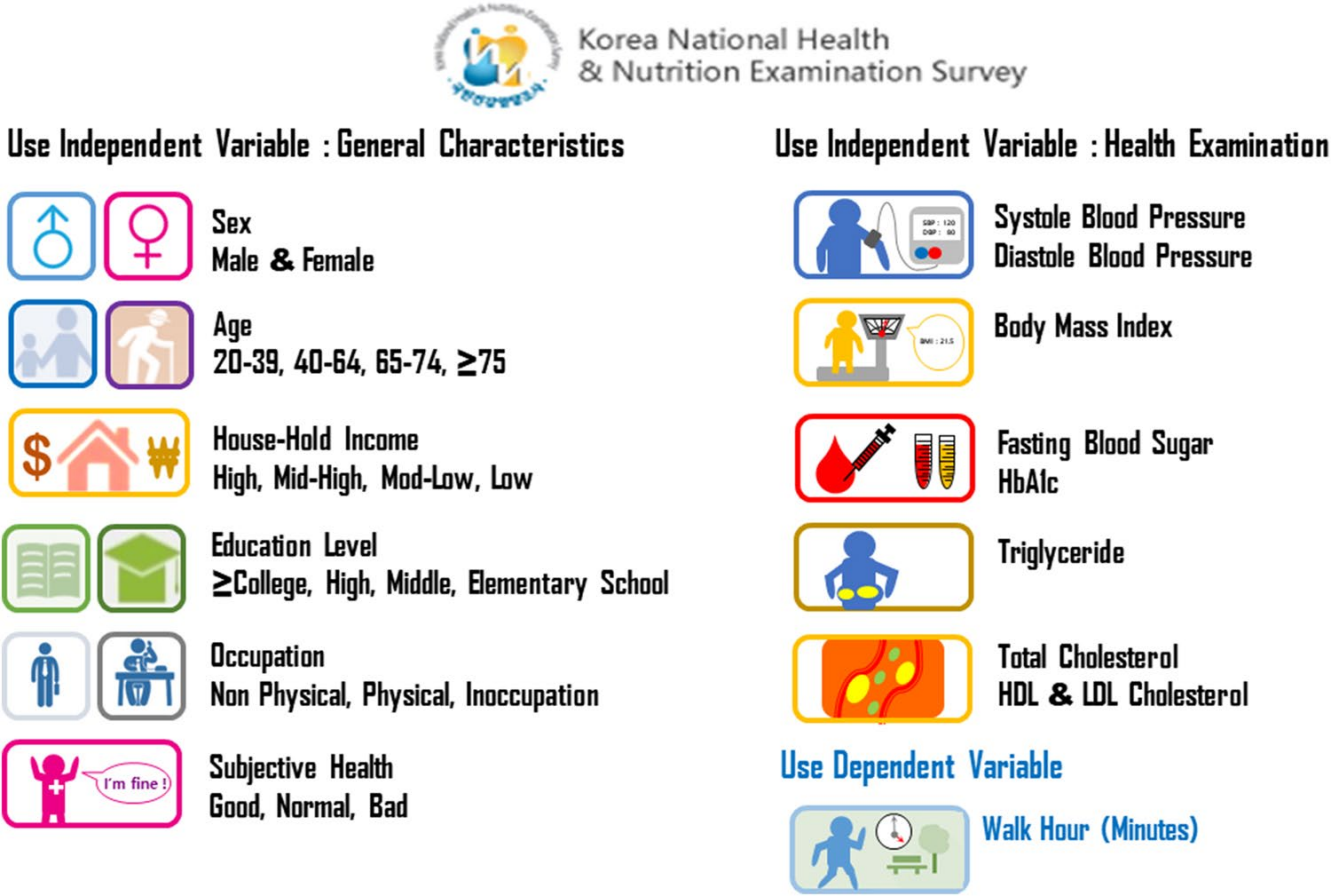
Research on composite data using national government statistics. We used a variable factor to analyze the changes in actual fine dust concentration, health behavior, index related health status and frequency of media reports related to fine dust. Republic of Korea National Health and Nutrition Examination Survey Data (KNHNES) for general characteristics, subjective health status and walking hour for dependent variable.

We analyzed the data from 2008–2017. The variables analyzed for the general characteristics were sex, age, house-hold income, education level, occupation group, and subjective health status. To assess health status, data on systolic blood pressure, diastolic blood pressure, body mass index, fasting blood sugar, Hemoglobin A1c (HbA1c), total cholesterol, high-density lipoprotein (HDL) cholesterol, and low-density lipoprotein (LDL) cholesterol were analyzed. The dependent variables were walking level and duration (in minutes). The average walking duration was measured for South Korea and the metropolitan city, Seoul (Fig. 2).

### Environmental statistics yearbook data

We used data from the Environmental Statistics Yearbook^20^, which included measurements of air quality and PM_10_ in South Korea. This data control is the based cities code merge the PM_10_ data in Korea National Health and Nutrition Examination Survey Data cities code. Therefore, average PM_10_ levels in a decade was calculated for the whole nation. The variables used were PM_10_ for the whole nation’s average and PM_10_ and PM_2.5_ for the metropolitan city, Seoul’s average.

### Ministry of statistics Korea city and province basic statistics data

Data from the Ministry of Korea City & Province Basic Statistics were used^21^. The data included the number of parks and sports sites. This variable was used to assess the relationship between the city environment and walking time.

### Ministry of statistics social survey data

We used data from the Ministry of Statistics Social Survey^22^ to assess safety consciousness and policy making and utilization of basic data for people. The data used included the awareness of social safety and fear of walking at night. The survey is conducted biennially, and the years included for this study were 2008, 2010, 2012, 2014, and 2016. Social safety was summarized using good safety line and bad safety line. The awareness of good social safety was indicated by ‘very safe’ and ‘often safe’, whereas the awareness of bad social safety was indicated by ‘often not safe’ and ‘very not safe’.

### Press foundation-news big data and analysis system: the big kinds-mass media exposure data

We used Press Foundation is open provide social mass media exposure data in news big data & analysis system: The Big Kinds^23^. We searched for information on PM_10_ mass media exposure using these keywords: ‘find dust’, ‘PM_10_’, or ‘Particulate Matter 10’, These keywords exposure to the South Korea. It’s exposure frequency through press company.

### Statistical analysis

We used the open source statistical software R version 3.6.0 for all statistical analyses. Descriptive frequencies were used to analyze general characteristics; descriptive statistics to analyze PM_10_ and PM_10_ Mass Media Exposure Frequency in South Korea and Seoul walking time (in minutes) and Seoul PM_10_ and Seoul PM_2.5_ and Social Environment (Number of Park & Number of Sport site), ANOVA post-hoc Scheffe test to the according to decade years. Multiple line charts were used to display the data. Multiple regression analysis was performed to identify factors affecting walking time.

## Results

### General characteristics

We were subjects total 20,605 persons in National Health and Nutrition Examination Survey decade data. Decade average number of subjects percentages sex were Male 54.1%, Female 46.0%, Age were 20–39 years 28.3%, 40–64 years 52.5%, 65–74 years 14.0%, more than 75 years 5.3%, House-hold income were high groups 28.1%, mid-high groups 27.0%, mid-low groups 26.1%, low groups 18.9%, education level were more than college 31.0%, high school 34.3%, middle school 11.7%, less than elementary school 22.9%, occupation type were non-physical job 36.1%, physical job 27.7%, inoccupation 36.2% and subjective health state were good 31.0%, normal 48.1%, bad 20.9 (Table 1).

**Table 1:** General characteristics.

Variable	2008	2009	2010	2011	2012	2013	2014	2015	2016	2017
	n (%)	n (%)	n (%)	n (%)	n (%)	n (%)	n (%)	n (%)	n (%)	n (%)
**Sex**										
Male	2,615 (42.0)	974 (49.7)	937 (49.1)	944 (49.3)	431 (56.0)	449 (60.8)	448 (63.5)	2,020 (43.4)	553 (60.0)	545 (66.7)
Female	3,609 (58.0)	985 (50.3)	971 (50.9)	969 (50.7)	338 (44.0)	289 (39.2)	257 (36.5)	2,630 (56.6)	369 (40.0)	272 (33.3)
**Age**										
20–39	2,109 (33.9)	786 (40.1)	750 (39.3)	738 (38.6)	145 (18.9)	164 (22.2)	153 (21.7)	1,172 (25.2)	201 (21.8)	172 (21.1)
40–64	2,802 (45.0)	905 (46.2)	933 (48.9)	971 (50.8)	454 (59.0)	429 (58.1)	391 (55.5)	2,305 (49.6)	504 (54.7)	463 (56.7)
65**–**74	920 (14.8)	204 (10.4)	207 (10.8)	186 (9.7)	127 (16.5)	108 (14.6)	114 (16.2)	770 (16.6)	146 (15.8)	120 (14.7)
≥ 75	393 (6.3)	64 (3.3)	18 (0.9)	18 (0.9)	43 (5.6)	37 (5.0)	47 (6.7)	403 (8.7)	71 (7.7)	62 (7.6)
**House-hold income**										
High	1,674 (26.9)	572 (29.2)	548 (28.7)	563 (29.4)	225 (29.3)	196 (26.60	184 (26.1)	1,379 (29.7)	258 (28.0)	222 (27.2)
Mid-high	1,634 (26.3)	570 (29.1)	533 (27.9)	563 (29.4)	194 (25.2)	206 (27.9)	187 (26.5)	1,283 (27.6)	223 (24.2)	208 (25.5)
Mod-low	1,635 (26.3)	471 (24.0)	520 (27.3)	534 (27.9)	222 (28.9)	178 (24.1)	191 (27.1)	1,1,369 (24.4)	244 (26.5)	196 (24.0)
Low	1,281 (20.6)	346 (17.7)	307 (16.1)	253 (13.2)	128 (16.6)	158 (21.4)	143 (20.3)	852 (18.3)	197 (21.4)	191 (23.4)
**Education level**										
≥ College	1,609 (25.9)	583 (29.8)	673 (35.3)	685 (35.8)	190 (24.7)	214 (29.0)	196 (27.8)	1,532 (32.9)	316 (34.3)	285 (34.9)
High School	2,117 (34.0)	737 (37.6)	683 (35.8)	720 (37.6)	266 (34.6)	256 (34.7)	232 (32.9)	1,540 (33.1)	281 (30.5)	265 (32.4)
Middle School	706 (11.3)	221 (11.3)	201 (10.5)	205 (10.7)	106 (13.8)	89 (12.1)	92 (13.0)	507 (10.9)	104 (11.3)	99 (12.1)
≤ Elementary School	1,792 (28.8)	418 (21.3)	351 (18.4)	303 (15.8)	207 (26.9)	179 (24.3)	185 (26.2)	1,071 (23.0)	221 (24.0)	168 (20.6)
**Occupation**										
Non-Physical	1,853 (29.8)	709 (36.2)	759 (39.8)	728 (38.1)	268 (34.9)	283,938.3)	245 (34.8)	1,666 (35.8)	332 (36.0)	302 (37.0)
Physical	1,905 (30.6)	518 (26.4)	506 (26.5)	507 (26.5)	216 (28.1)	211 (28.6)	210 (29.8)	1,139 (24.5)	259 (28.1)	230 (28.3)
Inoccupation	2,466 (39.6)	732 (37.4)	643 (33.7)	678 (35.4)	285 (37.1)	244 (33.1)	250 (35.5)	1,845 (39.7)	331 (35.9)	285 (34.9)
**Subjective health state**										
Good	2,605 (41.9)	861 (44.0)	707 (37.1)	697 (36.4)	202 (26.3)	180 (24.4)	179 (25.4)	1,356 (29.2)	229 (24.8)	164 (20.1)
Normal	2,045 (32.9)	658 (33.6)	865 (45.3)	920 (48.1)	419 (54.5)	408 (55.3)	373 (52.9)	2,361 (50.8)	475 (51.5)	460 (56.3)
Bad	1,574 (25.3)	440 (22.5)	336 (17.6)	296 (15.5)	148 (19.2)	150 (20.3)	153 (21.70	933 (20.1)	218 (23.6)	193 (23.6)
Total	6,224 (100.0)	1,959 (100.0)	1,908 (100.0)	1,913 (100.0)	769 (100.0)	738 (100.0)	705 (100.0)	4.650 (100.0)	922 (100.0)	817 (100.0)

Frequency test: categorical variables were expressed as sample number and %.

### Trends for walking time (minutes), PM_10_, and PM_10_ mass media exposure frequency in South Korea and walking time (minutes), PM_10_, and PM_2.5_ in Seoul City

**Figure 3:**
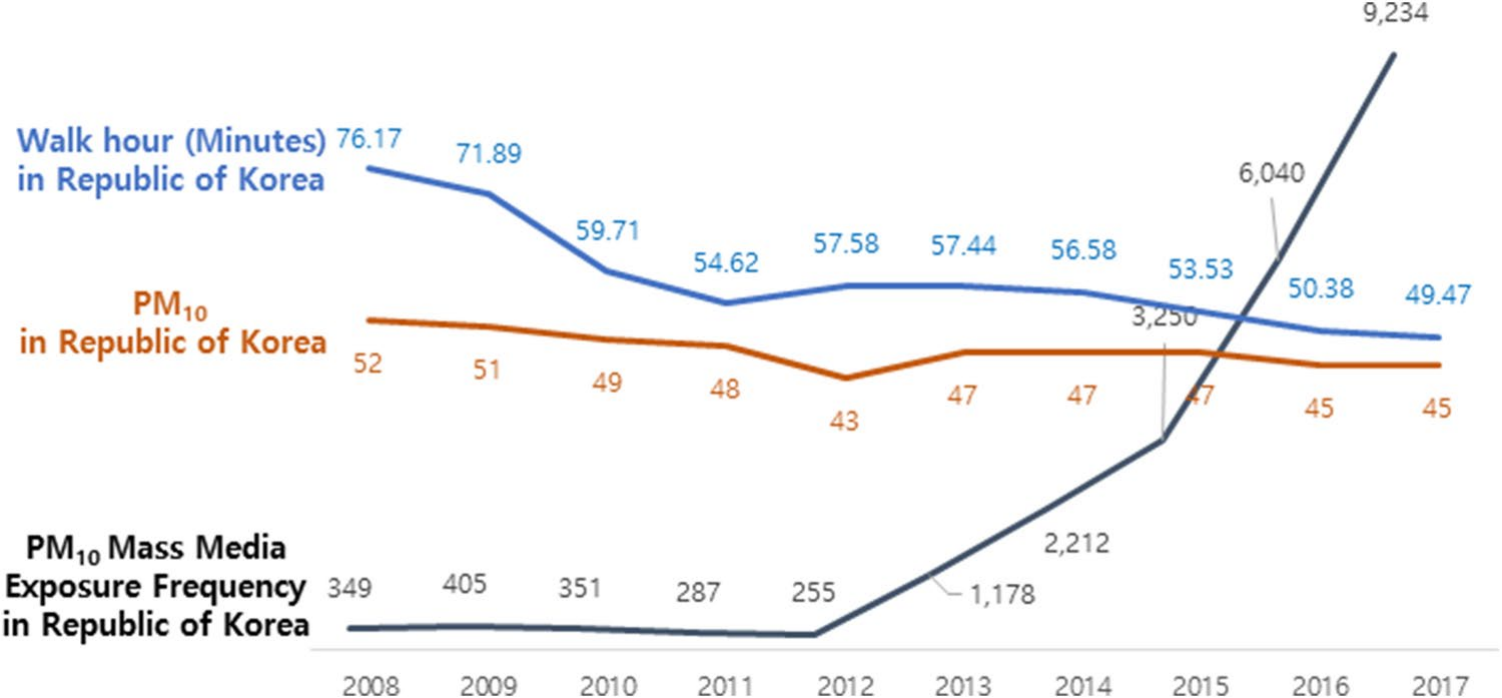
Trends for daily average walk time (minutes) of Republic of Korea & annual average concentration of PM10 (Particulate Matter 10) in Republic of Korea and frequency of annual average of mass media exposure related PM10 in Republic of Korea from 2008 to 2017. **Blue line.** The daily average walk-time (minutes) of the Republic of Korea’s public decreased from 76.17 min in 2008 to 54.62 min in 2011 and decreased steadily to 49.47 min in 2017. **Brown line**. The annual average concentration of PM10 in the whole nation of Republic of Korea decreased from 52 μg/m^3^ in 2008 to 45 μg/m^3^ in 2017. **Black line**. The annual average frequency of mass media exposure related to PM10 in Republic of Korea increased from 349 in 2008 to 9,234 in 2017.

**Figure 4:**
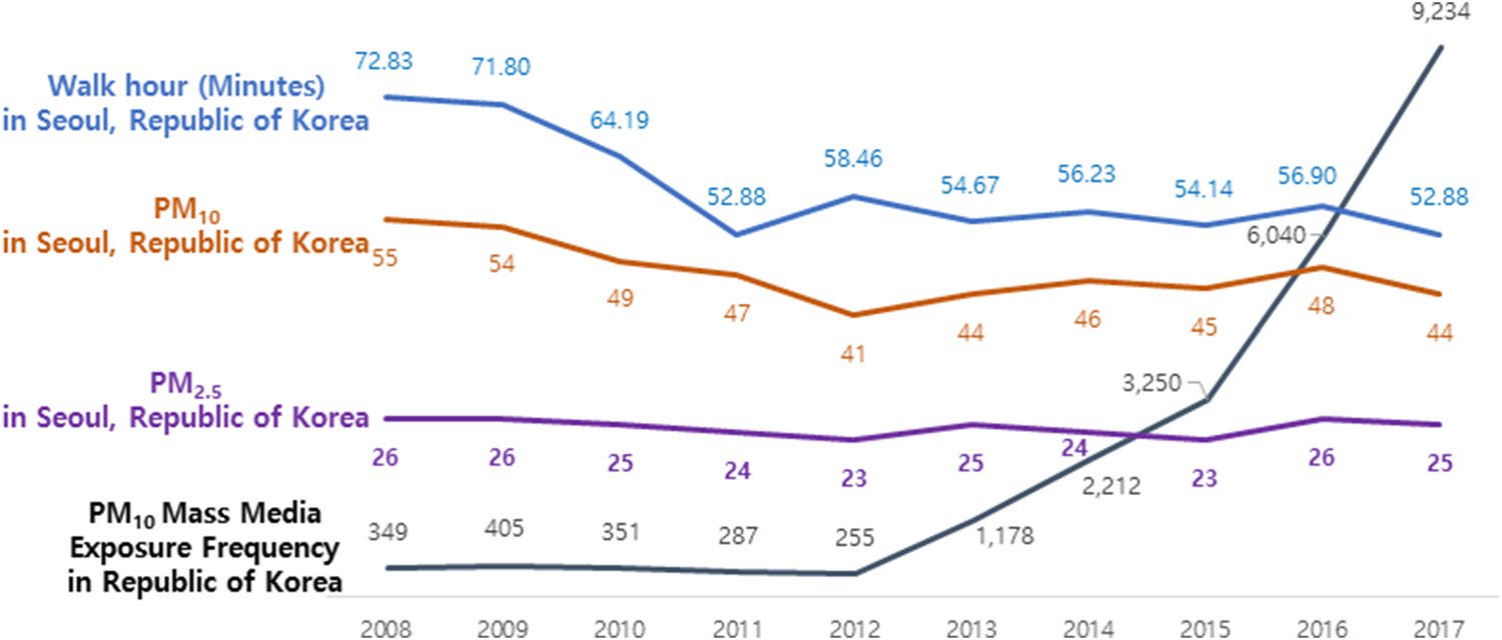
Trends for daily average walk time(Minutes) of in Seoul, Republic of Korea and annual average concentration of PM10(Particulate Matter 10) in Seoul, Republic of Korea & annual average concentration of PM2.5(Particulate Matter 2.5) in Seoul, Republic of Korea and frequency of annual average of mass media exposure related PM10 in Republic of Korea from 2008 to 2017. **Blue line.** The daily average walk-time(Minutes) of Seoul, Republic of Korea’s public decreased from 72.83 minutes in 2008 to 52.88 minutes in 2017. **Brown line.** The annual average concentration of PM10 in Seoul, Republic of Korea decreased from 55 μg/m^3^ in 2008 to 44 μg/m^3^ in 2017. **Purple line**. The annual average concentration of PM2.5 in Seoul, Republic of Korea not changed at years. **Black line**. The annual average frequency of mass media exposure related to PM10 in Republic of Korea increased from 349 in 2008 to 9,234 in 2017.

The average walking time (in minutes) decreased from 76.17 min in 2008 to 49.47 min in 2017. Concurrently, PM_10_ levels decreased from 52 μg/m^3^ in 2008 to 45 μg/m^3^ in 2017, but PM_10_ mass media exposure frequency increased from 349 in 2008 to 9,234 in 2017 (Fig. 3). Walking time (in days) decreased from 4.53 to 3.75 days in 2017 (Table 2). In Seoul, walking time (in minutes) decreased from 72.83 min in 2008 to 52.88 min in 2017. Concurrently, PM_10_ levels decreased from 55 μg/m^3^ in 2008 to 44 μg/m^3^ in 2017. However, the trends for PM_2.5_ did not change over the years (Fig. 4); walking time (in days) among Seoul residents repeatedly decreased and increased over the decade (Table 2).

**Table 2:** Decade years compare of walk hour (min) in Republic of Korea & Seoul walk hour (min).

Variable	2008	2009^b^	2010^c^	2011^d^	2012^e^	2013^f^	2014^ g^	2015^ h^	2016^i^	2017^j^	F (*p*)	Scheffe
	Year M (SD)	Year M (SD)	Year M (SD)	Year M (SD)	Year M (SD)	Year M (SD)	Year M (SD)	Year M (SD)	Year M (SD)	Year M (SD)		
Walk hour (min)	76.17(99.48)	71.89(88.22)	59.71(71.61)	54.62(54.62)	57.58(70.80)	57.44(64.91)	56.58(74.48)	53.53(61.95)	50.38(60.22)	49.47(61.09)	100.634(< .001)	a, b > c, d, e, f, g, h > I, j
Walk day	4.53(2.59)	4.31(4.31)	3.95(2.64)	3.81(2.62)	3.83(2.59)	3.76(2.54)	3.95(2.63)	3.85(2.61)	3.78(2.62)	3.75(2.63)	73.313(< .001)	a, b > c, d, e, f, g, h, I, j
Seoul walk hour (min)	72.83(97.21)	71.80(80.66)	64.19(71.63)	52.88(52.96)	58.46(58.69)	54.67(52.43)	56.23(62.10)	54.14(52.68)	56.90(61.64)	52.88(56.50)	17.029(< .001)	a, b > c, d, e, f, g, h, I, j
Seoul walk day	4.59(2.51)	4.44(2.43)	4.22(2.53)	4.14(2.50)	4.32(2.40)	4.17(2.47)	4.44(2.48)	4.60(4.44)	4.60(2.37)	4.59(2.40)	5.734(< .001)	a, b, h, I ,j > c. d. e > f

Continuous variables were expressed as M(SD).

M: year mean value, SD: standard deviation, ANOVA(F test): post-hoc method: Scheffe.

Significant *p* value: *p* < .05.

### Trends for number of park and number of sport site in South Korea

**Figure 5:**
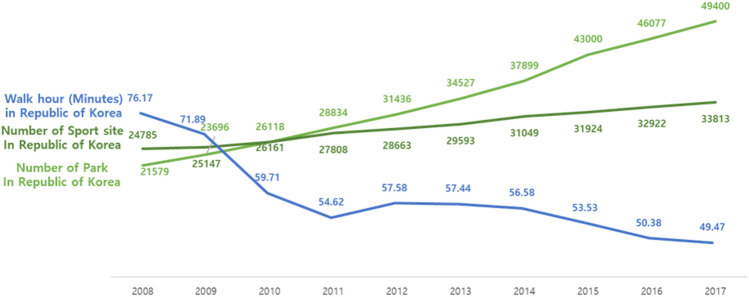
Trends for daily average walk time(Minutes) of Republic of Korea & annual number of Sport site in Republic of Korea and annual number of Park in Republic of Korea from 2008 to 2017. **Blue line.** The daily average walk-time(Minutes) of the Republic of Korea’s public decreased from 76.17 minutes in 2008 to 54.62 minutes in 2011 and decreased steadily to 49.47 minutes in 2017. **Green line**. The annual number of Sport site in Republic of Korea increased 24,785 in 2008 to 33,813 in 2017. **Light green line.** The annual number of Park in Republic of Korea increased 21,579 in 2008 to 49,400 in 2017.

The number of walking-friendly parks and sport sites increased in the decade from 2008 to 2017. The number of parks increased from 21,579 in 2008 to 49,400 in 201; whereas that of sport sites increased from 24,785 in 2008 to 33,813 in 2017 (Fig. 5).

### Trends for awareness of social safety and Afraid for night walk in South Korea

**Figure 6:**
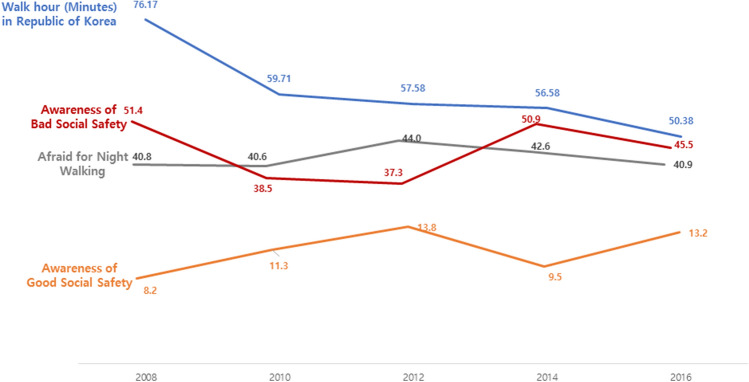
Trends for daily average walk time(Minutes) of Republic of Korea & Once response rate every 2 years about Awareness of bad social safety in Republic of Korea & Once response rate every 2 years about Afraid for Night walking in Republic of Korea and Once response rate every 2 years about Awareness of Good social safety in Republic of Korea, included 2008, 2010, 2012, 2014, 2016. **Blue line.** The daily average walk-time (minutes) of the Republic of Korea’s public decreased from 76.17 minutes in 2008 to 50.38 minutes in 2016. **Red line.** The response rate about Awareness of Bad social safety in Republic of Korea was decrease and increased repeatedly. **Gray** **line.** The response rate about Afraid for night walking not changed. **Yellow line.** The response rate about Awareness of Good social safety increased 8.2 percent in 2008 to 13.2% in 2016.

The awareness of social safety was described as either good safety line or bad safety line. Awareness of good social safety line increased whereas that of bad social safety line repeatedly decreased and increased. Trends on fear of walking at night did not change (Fig. 6).

### Trends for budget of health expenditure and budget of land and area development expenditure in South Korea

The nation’s budget on health, land, and area development was continuously increased. For health, the budget increased from 41,801 billion won (35 billion dollar) in 2008 to 45,011 billion won (37 billion dollar) in 2017. For land and area development, the budget increased from 64,417 billion won (54 billion dollar) in 2008 to 172,426 billion won (145 billion dollar) in 2017 (Table 3).

**Table 3:** PM10 and PM10 mass media exposure frequency in Republic of Korea and Seoul PM10 and Seoul PM2.5 and social environment in Republic of Korea.

Variable	2008	2009	2010	2011	2012	2013	2014	2015	2016	2017
	Year Mean	Year Mean	Year Mean	Year Mean	Year Mean	Year Mean	Year Mean	Year Mean	Year Mean	Year Mean
PM10*	52	51	49	48	43	47	47	47	45	45
PM10 mass media† exposure frequency	349	405	351	287	255	1,178	2,212	3,250	6,040	9,234
Seoul PM10*	55	54	49	47	41	44	46	45	48	44
Seoul PM2.5*	26	26	25	24	23	25	24	23	26	25
Number of park†	21,579	23,696	26,118	28,834	31,436	34,527	37,899	43,000	46,077	49,400
Number of sport site†	24,785	25,147	26,161	27,808	28,663	29,593	31,049	31,924	32,922	33,813
Awareness of good social safety	8.2	–	11.3	–	13.8	–	9.5	–	13.2	–
Awareness of bad social safety	51.4	–	38.5	–	37.3	–	50.9	–	45.5	–
Afraid for night walking	40.8	–	40.6	–	44.0	–	42.6	–	40.9	–

*PM10 and PM2.5 unit: μg/m^3^*.*

^†^Number of frequent.

Awareness of good and bad social safety and afraid night walking-unit: %.

### Trends for health examination in South Korea

Health Examination variables were systolic blood pressure, diastolic blood pressure, body mass index, fasting blood sugar, HbA1c, triglycerides, total cholesterol, HDL cholesterol, and LDL cholesterol. The average systolic blood pressure increased from 115.83 mmHg in 2008 to 124.13 mmHg in 2017. Diastolic blood pressure also increased from 74.70 mmHg in 2008 to 79.61 mmHg in 2017. Body mass index also increased from 23.61 kg/m^2^ in 2008 to 23.88 kg/m^2^ in 2017. Fasting blood sugar increased from 98.21 mg/dL in 2008 to 100.91 mg/dL in 2017. HbA1c increased from 5.36% in 2008 to 5.66% in 2017. Triglycerides repeatedly decreased or increased from 131.26 mg/dL to 130.28 mg/dL. Total cholesterol increased from 187.74 mg/dL to 211.94 mg/dL. HDL cholesterol decreased from 51.42 mg/dL to 42.18 mg/dL. LDL cholesterol increased from 111.38 mg/dL to 119.87 mg/dL (Table 4). The trends for health status examination has been bad in South Korea (Table 4).

**Table 4:** Decade years compare of health examination in Republic of Korea.

Variable	2008^a^	2009^b^	2010^c^	2011^d^	2012^e^	2013^f^	2014^ g^	2015^ h^	2016^i^	2017^j^	F (*p*)	Scheffe
	Year mean (SD)	Year mean (SD)	Year mean (SD)	Year mean (SD)	Year mean (SD)	Year mean (SD)	Year mean (SD)	Year mean (SD)	Year mean (SD)	Year mean (SD)		
Systole blood pressure	115.83 (17.26)	119.17 (16.69)	119.94 (16.83)	117.51 (16.80)	125.13 (16.38)	123.60 (15.91)	122.77 (15.14)	119.14 (17.16)	124.12 (15.12)	124.13 (16.28)	53.181 (< .001)	a < b, c, d, h < e, f, g, I, f
Diastole blood pressure	74.70 (10.96)	78.30 (10.59)	77.92 (10.67)	76.49 (70.37)	80.29 (10.85)	79.25 (11.14)	78.71 (9.88)	75.05 (10.17)	79.67 (10.41)	79.61 (11.15)	103.696 (< .001)	a, h < b, c, d, < e, f, g, I, j
Body mass index	23.61 (3.27)	23.66 (3.34)	23.57 (3.30)	23.66 (3.33)	23.74 (3.33)	23.77 (3.38)	23.66 (3.34)	23.91 (3.44)	23.97 (3.51)	23.88 (3.48)	9.449 (< .001)	a, b, c, d, e, f, g < j, I, j
Fasting blood sugar	98.21 (23.40)	97.39 (21.96)	97.20 (21.65)	97.71 (22.29)	98.48 (21.52)	98.96 (21.00)	99.42 (21.92)	100.87 (24.01)	101.31 (24.58)	100.91 (23.63)	29.067 (< .001)	a, b, c, d > e, f, g < h, I, j
HbA1c	5.36 (1.02)	5.37 (0.92)	5.39 (0.99)	5.68 (0.63)	5.74 (0.74)	5.84 (0.74)	5.75 (0.75)	5.66 (0.76)	5.66 (0.57)	5.66 (0.78)	156.636 (< .001)	a, b, c < d, e, f, g, h, I, j
Triglyceride	131.26 (93.43)	131.25 (91.45)	128.86 (88.15)	129.84 (88.23)	128.20 (83.91)	131.03 (90.39)	131.12 (90.75)	132.07 (89.97)	135.20 (92.51)	130.28 (87.48)	2.479 (.008)	c, d, j < a,b,f,g < h, i
Total cholesterol	187.74 (36.01)	186.62 (35.75)	187.70 (37.17)	188.73 (36.14)	208.43 (40.50)	204.76 (39.77)	203.96 (37.39)	190.41 (35.92)	210.15 (41.570	211.94 (42.53)	99.505 (< .001)	a, b, c, d < h < e, f, g < i, j
HDL cholesterol	51.42 (12.63)	52.33 (12.66)	52.89 (12.83)	53.20 (13.00)	42.41 (9.21)	43.84 (9.69)	43.70 (10.03)	50.98 (12.97)	41.95 (10.03)	42.18 (9.34)	127.024 (< .001)	a, b, c, d > h, > e, f, g, i, j
**LDL cholesterol**	−−	111.38 (31.13)	112.70 (31.77)	114.28 (32.80)	120.41 (35.25)	114.97 (34.76)	116.85 (33.59)	113.79 (31.92)	117.02 (35.57)	119.87 (38.14)	10.243 (< .001)	b, c, h < d, f, g < i, f < e

Continuous variables were expressed as mean (SD).

SD: standard deviation, ANOVA (F test): post-hoc method: Scheffe.

Significant *p* value: *p* < .05.

Systole and diastole blood pressure unit: mmHg, Body Mass Index = Body Weight/(Height^2^),

Fasting blood sugar and triglyceride and total cholesterol, high density lipoprotein(HDL) cholesteol & Low Density Lipoprotein(LDL) Unit: mg/dl, HbA1c(glycated hemoglobin) Unit: %.

### Factor affecting to walk time (min) in South Korea

In order to examine factors affecting walking time, data from 2008 to 2017 were analyzed using multiple regression analysis (step-wise). Variables that can affect walking time in the dataset: PM10, PM10 Mass Media Exposure, Awareness of Good Social Safety, Afraid for night walking, Number of sport site, Number of park, Sex, Age, House-hold income, Education Level, Occupation, and Subjective health state were included. As a result, House-hold income, Occupation, Number of sport site, and Number of park were excluded. As a result of controlling these variables, the walking time decreased by β = − .022 as the PM10 increased and β = − .053 as the PM10 Mass media exposure increased (Table 5).

**Table 5:** Factor affecting to walk time (min).

Variable	Walk time (min)	
	β	*p*
PM10	− .022	.005
PM10 mass media exposure	− .053	< .001
Awareness of good social safety	− .069	< .001
Afraid for night walking	− .025	.002
Sex—female (ref: male)	− .078	< .001
Age	− .052	< .001
Education level	− .057	< .001
Subjective health state	.047	< .001
Model fit	R^2^ = .021, F = 71.750, *p* < .001	

Multiple regression analysis (step-wise): exclude variable (house-hold income, occupation, number of sport site, number of park).

Whole analysis the dataset 2008–2017.

**β**: Standardized coefficient, *p*: *p* value.

## Discussion

This research was based on the fact that South Korea has a conducive physical environment (number of park, number of sport site, decreased PM_10_) and a friendly social environment (increased awareness of good social safety, increased budget of health, increased budget of land and area development) for walking. However, in the last decade, the national average time for walking has decreased. From ‘Fig. 3’, one would observe that the walking time (minutes) has decreased, whereas the PM_10_ mass media exposure frequency increased. At the same time, the health status of residents of South Korea have deteriorated according to health examination data, and this has been reasoned to be as a result of the reduction in walking behaviors. The beneficial effects of walking on health include the reduction in systolic blood pressure, diastolic blood pressure, triglycerides, total cholesterol, fasting blood sugar, and HbA1c^24–31^. Therefore, we presumed that the reduction in walking behaviors among residents of South Korea is as a result of the fear of increased mass exposure to air pollutants, such as PM_10_, which they think may expose them to some forms of danger. But this really factors the decreased PM10 decennary. And South Korea’s Average year PM_10_ was not danger and many improve the air quality than past 10 years, for the do not walking and do not health benefits. South Korea governments fighting making policy and finance the budget for improve air quality and good for healthy environment & good for social safety environment. This really problem is the press companies over exposure of the mass media output results that it. Examples reference of negative health affect mass media exposure, television and magazine were Internalization of the thin beauty ideal, social comparison, and activation of the thinness schema are clearly among the processes which mediate the effects of the media on body dissatisfaction, weight concerns and disordered eating behaviors32. Mass media was not every day positive affect. This influence of particulate matter risk perception in the public is reflected to various type of risk related environmental, social and economics^33-36^. You et al.^34^ said that the large amount of media reports on uncertain basis of the particulate matter frame affects the physical activity of the public. In others studies^37,38^ said that the public is perceived and more reacts about the risk of particulate matter due to increase of media coverage and warning message.

According to data from the Korean National Health and Nutrition Examination survey^19^, the average walking time of people aged above 19 years is decreasing year by year. The reasons for this are the lack of absolute walking time due to long working hours^39^, lack of ecological^40^ and suitable urban environment-facility^41^, the amount of health-related budget input for walking in the city or bicycles^42^, and awareness of city safety for living^43^. Furthermore, Various studies have reported that economic crisis affects health related behavior^44–46^. Studies that examined changes in health behaviors of Icelandic people due to the economic crisis indicated that the economic crisis is responsible for the reduction of health-compromising behavior and health-promoting behavior^44^, another study in Spain said that it caused health-related behavior changes due to economic-financial crisis^45^. On the other hand, as a result of analyzing changes in the health-related behavior of Greeks due to the financial crisis, physical activity increased during the first economic crisis in 2006 and the second economic crisis in 2011^46^. In the case of Korea, the crisis triggered in the US financial market in 2008 spread to the world, and the economic crisis began and continued in Korea. As a result, the psychological burden of the people was increased, consumer life was greatly reduced, and activities in all fields were stagnant^47,48^. In response to this economic crisis, the public focused on job search and alternative economic activities, showing a decrease in physical activity such as walking^47^. These suggest that the problem is not only a factor of the individual but also a factor of the various ecological backgrounds that can affect the walking time^49,50^. In our study, walking time decreased in spite of the increase in ecological urban sites and the positive awareness of unban safety (Table 3). If this is the case, then the cause of the decrease in walking time is likely a factor of the change in the individual perception level. Therefore, the change in perception was attributed to an increase in awareness of the crisis due to increased media reports related to fine dust in this study. A study by Kim et al.^51^ conducted a frame analysis and a source analysis of a fine dust reporter to examine the risk perception of fine dust. During the frame analysis, it was found that the amount of articles reported on an uncertain basis was quite large, and the most frequently used experts as sources of information. In addition, Kim et al.^52^ said that communication and media studies have a profound effect on the people living in modern times, and that a large amount of articles reported as an uncertain basis during the analysis of the fine dust frame affects people's physical activities. Also, people are increasingly aware of the dangers of particulate matter due to increased media coverage and warning messages^37,38^.

PM risk perception is not only a real risk to health, but also a complex phenomenon affecting changes in public opinion and policy^53^. For example, Outdoor activity such as walking are limited by the level of particulate matter^54^. The limitation of these outdoor activity brings about the increase in economic burden due to the use of defensive products, such as mask or air filters, and the increase of alternative service to replace the traditional outdoor activity, expansion of indoor activity, such as yoga and weight training^55,56^. In addition, South Korea is a country with very well-developed Internet and Social networking service and particulate matter risk perception is formed depending on the frequency of media reports regardless of the actual frequency of occurrence and the risk of harm to health^57^. In a summary, South Korea’s particulate matter-related policies should take into account not only economic development but also social point of view^58^.

These type of media report which address above, for example, “The inflow of fine dust from China increased up to 86%”^59^ as a foreign factor, gave the people a sense of ‘fine dust = China’. However, this was only the result of the analysis of a certain period of China’s influence. In fact, the concentration of fine dust in most areas of China has decreased, but the uncertainty of the increase in fine dust concentration due to the relocation of a specific industrial complex in China has increased the public’s fear about fine dust^59^. Even if it is not solely the risk of fine dust created by media coverage, the media’s influence on perception of risk factors related fine dust cannot be ignored^60^. Previous reports^15^ indicated that the concentration of fine dust in Korea has been on a declining trend since 2007, but only slightly increased in 2013. In Kim's study^15^, as reported in our research, the news of fine dust-related media rapidly increased from the year 2013, and the perceived risk that did not actually exist tends to be misconstrued as a risk issue due to the explosive increase in media coverage. In addition, despite the fact that these studies are media reports related to fine dusts, there has been an increase in the number of reports for the purpose of promoting and selling specific products. The problem with these reports is that they can provide distorted information to achieve commercial purposes rather than present solutions to effectively address such risk situations as fine dust.

Fine dust is very small in size and enters the body through the breath of man, accumulates, and causes various health problems^61^. Many studies have reported that exposure to micro dust promotes mortality-related illnesses, such as changes in blood pressure, respiratory diseases, cardiovascular diseases, and stroke^62–64^. Other studies have reported that, as well as health problems causing damage to the industry, such as primary industries, transport, and manufacturing^65^. Due to these negative effects, the public interest and concern about fine dust increases with the passage of time. To do this, accurate research and policy should be established so that the public can understand and judge correctly, without explaining the causes of the risk factors, not only in the media but also in government and academia. Min^66^ proposed the countermeasures of each institution for effective resolution of fine dust. First, the government should establish a solution through an integrated approach involving ministries. Second, the communication between the ministries and the public should be more open than the existing methods and it is necessary to improve the current administrative procedures and customs which may be obstacles to the identification of risk factors. Kim et al.^57^ said that in South Korea, where the Internet and social networking service is very well-developed, the public perception is formed almost by the frequency of media reports regardless of the actual concentration and frequency of particulate matter and its harmful effects on health. In addition, some studies^67,68^ show that the public overexposed to fragmented media reporting environmental stress factors such as particulate matter^69^ negatively aware of risk related health by emphasizing the role of communication for the public.

Third, it is suggested that academia should have a consciousness to solve the problem of air quality pollution, and should be able to converge various opinions based on an open research climate. Finally, the media should be able to fulfill the positive role of the media through fair reporting, devoid of the indulgence of irritating themes, and to be able to wait for "the right time" of academics and government. The government's interpretation and reflection of the policy should not lose the sense of the cold report^66^.

The limitations of our study are as follows. First, we did not control for bias from variables that could influence the correlation between micro dust media footprints and walking time reduction. The data were analyzed by two approaches, which were gradually based on the information provided by public authorities to determine their relevance; it seems a missed approach and the nature of the difficulty of interesting items of secondary materials. In addition, the reliability and validity of the analysis depends on the ability of the researcher to select the necessary information in the secondary data^70^. Seconds, we could not determine the actual effects of decreased walking time on human health and health effects in detail. This is also as a result of the limitation from the use of secondary data, which seems to be a missing item of interest. Despite these limitations, our study utilized a variety of secondary materials from credible public institutions that had conducted possible large-scale investigations, which were not limited to simple health data, weather, media reports, the government budget, but included combined and the bias was reduced.

## Conclusion

This research question was whether the decreasing walking time of the Korean people was really related to the increase in the concentration of fine dust. It was found that various variable affect the walking time of the Korean people rather than the concentration of fine dust actually increasing. Especially, the number of media related to fine dust affected the walking time of Korean people rather than the actual concentration of fine dust. We conclude that the increase in indiscriminate media reports without identifying various internal and external factors of risk factors, such as explosive increase in fine dust concentration, could be the cause. In addition, by analyzing various variables, such as the number of health behavior practice sites (number of parks, number of sport sites), awareness of social safety, and afraid of night activities, related to changes in walking time and health status during this period, it was concluded that a decrease in physical activity could have a negative impact on health status. Therfore, these phenomena are not not temporary or rapid, as our results. So, this study hope to call attention of all walks of life for the citizens of the Republic of Korea to change health-related behaviors.
